# Identification and characterization of sodium and chloride-dependent gamma-aminobutyric acid (GABA) transporters from eukaryotic pathogens as a potential drug target

**DOI:** 10.6026/97320630014021

**Published:** 2018-01-31

**Authors:** Benson Otarigho, Mofolusho O Falade

**Affiliations:** 1 Department of Biological Science, Edo University, Iyamho, Edo State; 2 Nigeria Cellular Parasitology Programme, Cell Biology and Genetics Unit, Department of Zoology, University of Ibadan, Ibadan, Nigeria; 3 Department of Molecular Microbiology & Immunology, Oregon Health & Science University, Portland, OR 97239, USA

**Keywords:** GABA transporters, eukaryotic pathogens, chemotherapy targets, parasites genomes

## Abstract

We explored 285 completed eukaryotic pathogen genomes for GABA transporter proteins as effective chemotherapy targets. We identified 8 GABA proteins that spread across 4 phyla with 5 different pathogen species; Eimeria mitis Houghton, Neospora caninum Liverpool, S. mansoni, S. haematobium and Trichinella spiralis. Sub-cellular localization prediction revealed that these proteins are integral membrane and are mostly insoluble. It is found that about 81% of these proteins are non-crystallizable and 15% are crystallizable. Transmembrane helices predictions show that the GABA transporters have 10, 11, 12 and 14 TMHs with 15, 23, 31 and 11%, respectively. It is further observed that most of these GABA transporters are from several parasites`genomes.

## Background

Infectious Pathogens are the main enemies of mankind from time immemorial [1, 2]. These pathogenic organisms cause different diseases to man and animals [2, 3]. Some of these diseases include malaria, trypanosomiasis, leishmaniasis, schistosomiasis, Cryptosporidiosis, Onchocerciasis and many more [3, 4, 5, 6]. More than 25% of humans die annually as a result of these diseases and about 50% of such deaths occur among the poorest countries of the tropic and subtropical regions of the world [7, 8]. Besides, most of these pathogens infect both domestic and wild animals, consequently, leading to zoonosis [9, 10, 11]. The recent dramatic increase in emerging infectious diseases among the human population has implicated some wildlife and domestic animals as an important source of most novel and dangerous pathogens [2, 6]. These animals are the influencing factor in the human infectious disease transmission cycle [2, 12]. Although, drugs have been developed against most of these infectious diseases, the emergences of resistant strains of some of the pathogens [13, 14] make control difficult. Besides, there are no vaccines for most infectious diseases [2, 8]. Therefore, there is need to develop alternative chemotherapy to supplement/complement the existing ones.

One of the recommended approaches in search of next generation therapeutic drugs is to explore available parasite genomes [15, 16]. Moreover, proteins that play vital roles in the nervous system have been suggested to hold promises for druggable target [16, 17]. The nervous system coordinates many vital functions for the parasite survival and reproduction, including host attachment and penetration, motor activity and migration, feeding and excretion, pairing, and egg laying [17, 18]. Some of these parasite nervous systems such as schistosomes are well developed and have a rich diversity of neurotransmitters such as Gamma-Amino Butyric acid (GABA), which inhibits nerve transmission [16, 17, 18, 19]. Chemotherapeutic drugs that target GABA act on the neurotransmitter by binding to glutamate-gated chloride channels in nerve and muscle cells of invertebrates including eukaryotic parasite [20, 21]. However, these drugs have little side effects on the respective host due to the fact that GABA receptors occur only in the mammalian central nervous system (brain and the spinal chord). This central nervous system is protected by the blood-brain barrier that prevents microscopic and large molecules to get into the brain [22, 23]. Consequently, these GABA drugs are much less toxic to mammals than to parasites, which lack such barrier [24]. This is the major reason why GABA drugs are much more safer to use in the treatment of infectious disease in man, livestock and pets. Consequently, GABA drugs are highly recommended for the treatment and control of infectious diseases [21, 25].

Recently, GABA has been investigated and found in a wide range of organisms including bacteria, fungi, higher plants and animals [26, 27, 28, 29]. Few literatures have actually explored the eukaryotic pathogen genomes to identified neurotransmitters for chemotherapy. Among the few is the work of Fuks and Coworker, [30] which explore the GABAergic signaling by linking it to a hypermigratory phenotype in the dendritic cells infected by T. gondii, as well as the review of Ribeiro and Patocka, [16] that clearly points out neurotransmitter transporters in schistosomes for drug discovery. Recently, publically accessible sequenced parasite genomes data and computational tools have enhanced the development of novel and alternative chemotherapy targets [31], therefore bridging the gap between scientific research and clinical application [32].

In the present work, we identified GABA transporter from different eukaryotic pathogen genomes. The identified proteins were structurally and functionally characterised using computational approach as well as looking at the evolutionary relatedness. The findings in this study may offer new and alternative possibilities for potential drug development against most parasitic diseases affecting both man and animals.

## Methodology

### Genome Analysis, Sequence Alignments

We thoroughly searched for gamma-aminobutyric acid, GABA, transporter using “GABA transporter” as bait on the recent version of EupathDB (http://eupathdb.org/eupathdb/) that consist of about 285 organisms` genomes [33]. The identified GABA transporter proteins were fetched and added to EupathDB basket. The fasta formats of the sequences were downloaded. Other public databases such as NCBI (http://www.ncbi.nlm.nih.gov) [34], GeneDB (http://www.genedb.org/Homepage) [35], Uniprot (http://www.uniprot.org) [36] and SchistoDB (http://schistodb.net/schisto/) [37] were also searched for eukaryotic parasites GABA transporter proteins. To confirm the novelty of our identifications, we did a literature search for the different parasite GABA transporters using google.scholar (https://scholar.google.com) [38] and Pubmed (http://www.ncbi.nlm.nih.gov/pubmed). To have good comparison, we included free-living organisms` GABA transporters; lower plants (Aspergillus nidulans, Chromera velia, Coprinopsis cinerea, Saccharomyces cerevisiae, Vitrella brassicaformis), green plants (Arabidopsis thaliana and Brassica napus), invertebrate (Bathymodiolus septemdierum, Crassostrea gigas and Bombyx mori) and vertebrate (H. sapiens) animals.

### Structural and functional properties prediction and annotation

In order to have good knowledge of the obtained GABA transporters, we subjected them to various physical and chemical parameters predictions. These parameters include the molecular weight, theoretical pI, amino acid composition, atomic composition, extinction coefficient, estimated half-life, instability index, aliphatic index and grand average of hydropathicity (GRAVY) for the proteins using a webserver tool, ProtParam (http://web.expasy.org/protparam/) [39]. The presence of signal peptide and the position of each sequence were checked using Signal P web tool (http://www.cbs.dtu.dk/services/SignalP/) [40] and target P (http://www.cbs.dtu.dk/services/TargetP/) [41]. Solubility status of the proteins was computed using PROSO [42]. Prediction of transmembrane helices was done by TMHMM Server v. 2.0 (http://www.cbs.dtu.dk/services/TMHMM/) and validated using CCTOP webtool (http://cctop.enzim.ttk.mta.hu/?=/jobs/submit) [43]. After which the 2 D format of the CCTOP transmembrane helices images were obtained. An in-silico prediction of protein crystallization propensity was done on each protein using CRYSTALP2 webserver (http://biominews. ece.ualberta.ca/CRYSTALP2.html) [44]. Some of these were confirmed and validated using other webtools such as Compute pI/Mw, (http://web.expasy.org/compute_pi/) [45, 46] to validate theoretical pI and molecular weight and AACompIdent (https://web.expasy.org/aacompident/) to validate the amino acid composition. Subcellular localization of each protein was predicted using an advanced protein subcellular localization prediction tool; WoLF PSORT (http://www.genscript.com/wolf-psort.html) [47].

### Phylogenetic tree and Evolutionary relatedness analysis

All phylogenetic trees were constructed using MEGA version 5.2 software [48]. Briefly, the protein sequences were copied and pasted onto the MEGA alignment explorer window and without gab. The sequences were aligned using clustalW, with all parameters at default settings and the alignment file was activated for phylogenetic analysis. Neighbor-joining method was first employed to analyze the phylogenetic tree and computation was done using the Poisson correction method [49] having the units of the number of amino acid substitutions per site.

Secondly, the evolutionary history was inferred by using the maximum likelihood method based on the equal input model [50]. The percentage of trees in which the associated taxa clustered together was also computed next to the branches. Neighbor-Join and BioNJ algorithms to a matrix of pairwise distances estimated using a JTT model, and then selecting the topology with superior log likelihood value was employed. Both trees were drawn to scale, with branch lengths measured in the number of substitutions per site. In all the analysis involved were 26 amino acid sequences and all ambiguous positions were removed for each sequence pair. There were a total of 1521 positions in the final dataset. The percentage of replicate trees in which the associated taxa clustered together in the bootstrap test were 1000 replicates [51]. To have comparison of all the TMHs across the proteins, a second maximum-likehood phylogenetic tree was constructed following the method describes and the CCTOP images were aligned side-by-side as shown in Figure 3.

After each evolutionary history from MEGA, the tree files in newick format were exported and visualised in the FigTree software version 1.4.2 for proper annotation. Features like the scale bar, bootstrap values and branch length coloration base on strength were selected and adjusted. In addition the node shapes and legends of these colour strength were also added. Each tree was exported in JPEG format. We went further to estimates base composition bias difference and evolutionary divergences that may occur between sequences using the same version of MEGA, in order to confirm relationship between the proteins. Analytical method, poisson model, uniform rate and complete deletion were selected for estimate variance, substitution model, rate pattern and data subset respectively. The results were exported in excel format.

## Results

Our thorough search for GABA transporters across the different genomics and proteomics database that are publically available revealed that these proteins could be found in 8 eukaryotic pathogens (Table 1) that can cause disease in man and animals. These pathogens include Eimeria mitis Houghton, Neospora caninum Liverpool, S. mansoni, S. haematobium and Trichinella spiralis, which spread across 5 species and 4 phylla. The plant pathogen GABA transporter added to this study was Fusarium graminearum. The S. haemtobium, S. mansoni and other parasite GABA transporters were obtained from SchistoDB, GeneDB and EupathDB respectively. Homo sapiens GABA transporters were included in all analyses to have a comparative view of these parasites. S. haematobium among the parasites has the highest number of identification. Human GABA transporters were obtained from NCBI database. After literature search for the novelty, we find out that most of these GABA transporters were identified for the first time in this work.

**Figure 1:**
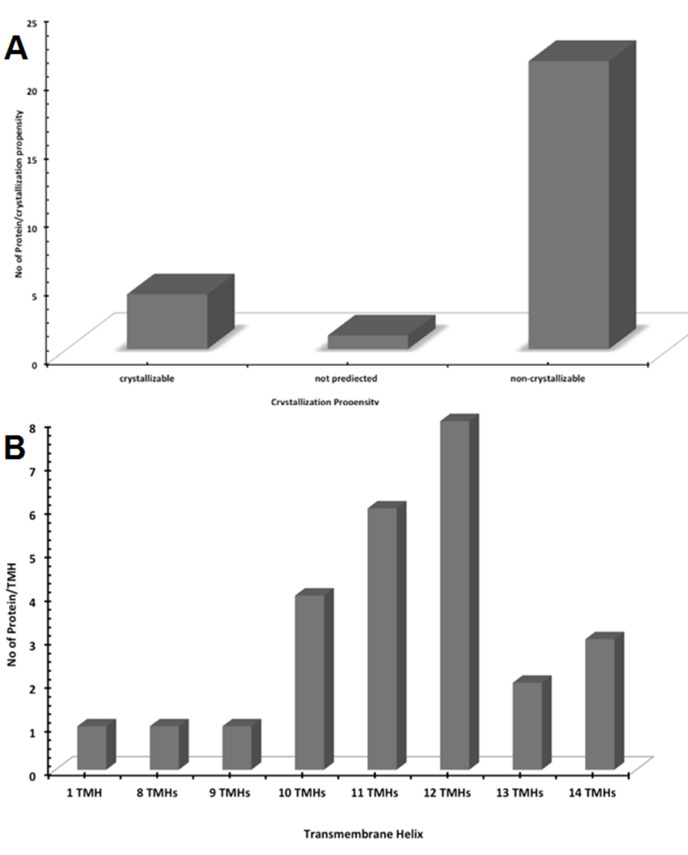
Sodium and Chloride-dependent Gamma-aminobutyric Acid Transporters showing [A] crystallization propensity; about 15% and 81% of the proteins were predicted to be crystallizable and none-crystallizable respectively and [B] number of transmembrane helices, which shows that that the most of the GABA transporters have 10, 11, 12 and 14 TMHs with 15, 23, 31 and 11% respectively.

The physical and chemical parameters computed for these proteins are presented in Table 1. Some of the physiochemical parameters analysed for each protein were number of amino acids, molecular weight, theoretical pI, total number of negatively charged residues, total number of positively charged residues, molecular formula, extinction coefficients (M-1 cm-1, at 280 nm measured in water) assuming all pairs of Cys residues form cystines and assuming all pairs of Cys residues form cystines, aliphatic index, grand average of hydropathicity (GRAVY), signal P, target P, TMHs, solubility, crystallization, propensity and sub-cellular localization. Our initial and confirmatory prediction shows that none of the identified proteins have signal peptide. All the protein were predicted to be insoluble during laboratory preparation in solvent except for Chromera velia with accession no; Cvel_21181. About 15% of the identified proteins were predicted to be crystallizable, while 81% are not none-crystallizable (Figure 1A). One of these proteins, Neospora caninum Liverpool with accession no; NCLIV_003090, was unable to be predicted because the tools available cannot take protein with too long sequences. All the pathogen proteins were predicted to be none-crystallizable (Table 1). When identified GABA transporters were computed for sub-cellular localization, we observed that they are integral membrane proteins, except for Eimeria mitis Houghton with accession no; EMH_0037150 that was predicted to be cytoplasmic and can be secreted. The sub-cellular localization prediction was automatically done based on the localization of 32 sequences similar proteins. SWISS-PROT and Gene ontology (GO) gave the confirmation prediction with high percentage identity. TMHs predictions show that the GABA transporters have 10, 11, 12 and 14 TMHs with 15, 23, 31 and 11% respectively (Figure 1B). A preliminary prediction had pointed out that these GABA transporters could be functioning as voltage-gated potassium/sodium channel complex in term of mechanism, though details are not given due to the poor 3D structure modeling.

**Figure 2:**
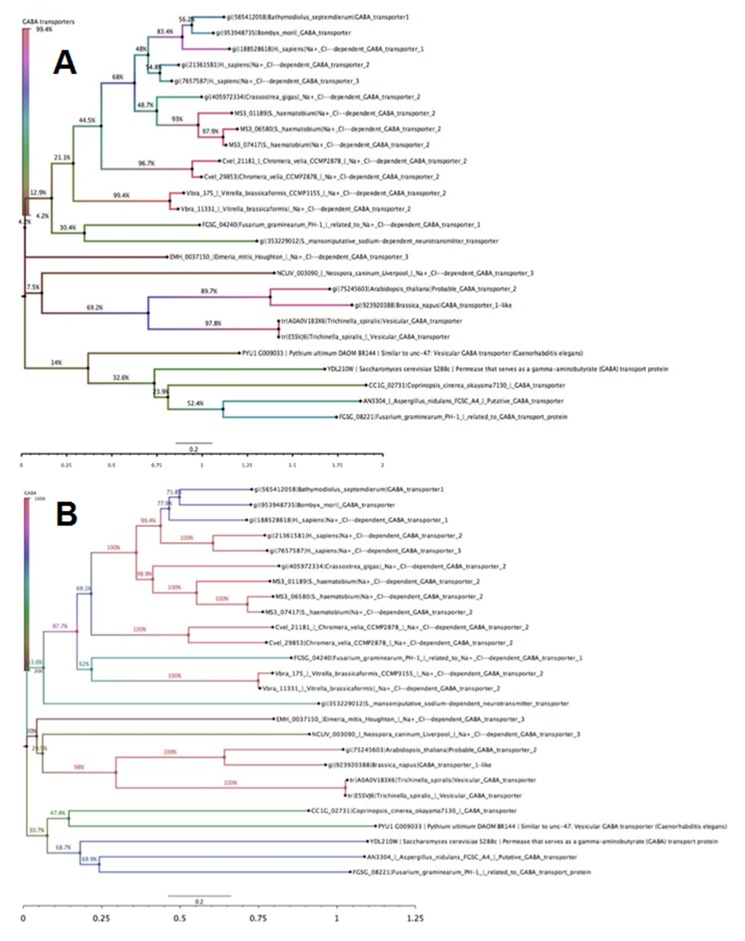
Phylogenetic tree of Sodium and Chloride-dependent Gamma-aminobutyric Acid Transporter proteins constructed using [A] Neighbor joining tree and [B] Maximum likelihood. In both phylogenetic tree methods, two of the three human GABA transporters are on the same minor clades, while all these proteins are on the same major clades with Bombyx mori GABA transporter and Bathymodiolus_septemdierum GABA transporter.

**Figure 3:**
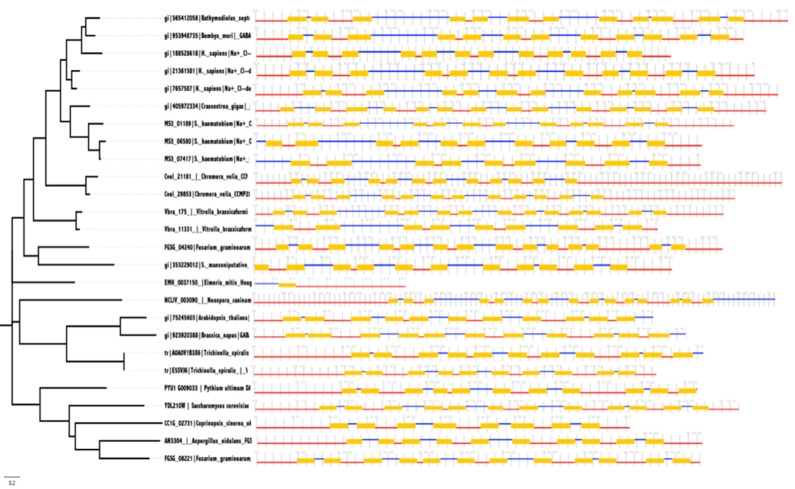
2D Transmembrane helices of Sodium and Chloride-dependent Gamma-aminobutyric Acid Transporter proteins arranged side by side against phylogenetic tree.

The detail analyses of the different (constructed based on maximum likelihood and neighbor joining) trees are shown in Figure 2A. The quantitative evolutionary relatedness of these GABA transporters is presented in Table 2. From the maximumlikelihood phylogenetic tree, two of the three human GABA transporters are on the same minor clades, however all these proteins are on the same major clades with Bombyx mori GABA transporter and Bathymodiolus_septemdierum GABA transporter. This shows that H._sapiens Na+ Cl-dependent GABA transporter 1 could be more closer to Bombyx mori and Bathymodiolus_septemdierum GABA transporter than H._sapiens Na+ Cl-dependent GABA transporter 2 and 3. We noticed that all the S. haematobium GABA transporters clustered together, however, S._haematobium Na+_Cldependent_ GABA_transporter_ (accession no; MS3_06580) and S._haematobium Na+_Cl- dependent GABA transporter (accession no; MS3_07417) are the closest. Crassostrea_gigas Na+_Cldependent GABA transporter 2 shared the same major clade with S. haematobium GABA transporters. The two proteoforms of Na+_Cl-dependent GABA transporter in Vitrella_brassicaformis, Chromera_velia and Trichinella spiralis are in the same clade each with high bootstrap values. Surprisely, Fusarium_graminearum Na+ Cl- dependent GABA transporter 1 (accession no, FGSG_04240) did not in any way relate to F._graminearum GABA_transport_protein (accession no, FGSG_08221). Rather, the formal is on the same clade with S. mansoni GABA transporters and the latter is in a clade with Aspergillus_nidulans putative_GABA_transporter and others. GABA transporter in Eimeria mitis Houghton and Neospora caninum Liverpool are different from the other proteins. When we analyzed the phylogenetic tree constructed based on the neighbor joining method (Figure 2B), similar observation was made. Moreover, the estimated values for evolutionary divergence between the GABA transporter sequences presented in Table 2, strongly supports both phylogenetic trees already discussed.

## Discussion

At present, developments of chemotherapeutic drugs focus on four main types of molecular targets, which include enzymes, receptors, ion channels and transporters [52, 53, 54]. Among these membrane proteins are mostly targeted with 60-70% of drugs developed towards infectious diseases [53, 55]. Moreover, more researches are focusing on membrane proteins such as ligandgated ion channels (LGICs) for the next generation drugs to eradicate these diseases [56]. GABA transporter, one of the most important LGICs, plays key roles in rapid synaptic transmission when bound to a ligand such as a neurotransmitter, which controls signaling and homeostasis [57, 58, 59]. These parasite proteins are the new directions for future research for the next generation chemotherapy of most infectious disease [56]. In addition, most of these eukaryotic pathogen genomes predict a rich diversity of neuro-receptors [15]. Therefore, we set out to first identify GABA transporter from known eukaryotic pathogen genomes that are publically available. These proteins were structurally and functionally characterised.

In this study, we find out that GABA transporters are spread across a wide range of eukaryotic pathogens species, which include S. haematobium, S. mansoni, Trichinella spiralis, Eimeria mitis Houghton, Neospora caninum Liverpool and others. This study revealed that this putative transporter is in different organisms and has conserved physiological functions. Other researchers made similar observation on bacterial ATP-binding cassette systems in different organisms [60, 61, 62]. Our results show that many of these identified putative Na+Cl- dependent GABA transporters are not yet fully annotated in available pathogen databases, other workers had reported similar observation [63]. Even the ones that are fully annotated have not been explored for clinical consideration, so this study also unveils many novel parasite GABA transporters, which have clinical implication in designing and development of new drugs. Our result also demonstrated that all except the Eimeria mitis Houghton GABA transporter is integral membrane protein. Moreover, these proteins are predicted to have at least 8 TMHs, which show they are permanently attached to the cell membrane.

The evolutionary relatedness computed for the identified proteins suggests that they may not be too different from another in terms of function and mechanism. S mansoni and S. haematobium GABA transporters are closely related and are also related to human GABA transporter 2. Bombyx mori GABA_transporter is closely related to Bathymodiolus septemdierum. The extent to which these parasite GABA transporters are related or different from that of human GABA transporters is presented in Table 2. This evolutionary relatedness of some of the proteins is also presented in the phylogenetic trees. This result shows that chemotherapeutic drugs that are effective on a given parasite could be promising against another related parasite. Most of the genes coding for these transporters may have underwent duplication event, which created a copy of every genomic region [64]. Over evolutionary time, many of the duplicated genes may have been lost through fractionation process [65]. However, others duplicated genes may have been retained in duplicate and their collinear arrangement as observed in the Figure 1.

Our evolutionary and phylogenetic analyses seem to give clues why drugs that target neurotransmitter are toxic to a wide range of related parasite. Moreover, since there is a growing concern about the efficacy of schistosomiasis only single drug of choice, praziquantel, and emergence of resistant strain of the pathogen, there have been focuses of GABA transporters of S. mansoni development of new therapeutic drugs [18, 66, 67] due to the important of the nervous system in the survival and reproduction of Schistosoma parasite [68].

This study also identified GABA transporters from the genome of Neospora caninum Liverpool. This membrane protein could also be targeted for chemotherapy in neosporosis, since available drugs such as clindamycin and other antiprotozoan such as are species and stage specific [69, 70]. Besides, the vaccines available are either too expensive or have mixed results when tested [70, 71, 72].

## Conclusion

In the search for novel and alternative chemotherapy of most infectious diseases pharmacologic manipulation of the GABA system and GATs may provide the means to achieve effective treatment. Our study successfully identified most of these parasite GABA transporters for the first time from parasite genomes that are publically available in known databases. These proteins were characterized by subjecting them to phylogenetic analyses. The findings in this study suggest that GABA transporters could offer new and alternative possibilities for potential drug development against most parasitic diseases affecting both man and animals.

## Conflict of Interests

The authors declare that there is no conflict of interests.

## Figures and Tables

**Table 1:** The Identified Sodium and Chloride-dependent Gamma-aminobutyric Acid (GABA) Transporters and their features

S.No	Organism	Acession No	No. of amino acids	Molecular weight	Theoretical pI	Total number of negatively charged residues	Total number of positively charged residues	Total number of atoms	Extinction coefficients(M-1 cm-1, at 280 nm measured in water) assuming all pairs of Cys residues form cystines	Extinction coefficients(M-1 cm-1, at 280 nm measured in water) assuming all pairs of Cys residues form cystines	Instability index	Aliphatic index	Grand average of hydropathicity (GRAVY)	Signal P	Target P	TMHs	SOLUBILITY	Crystallization propensity	Sub-cellular localization
1	Aspergillus nidulans	AN3304	517	56699.6	7.66	32	33	7992	105225	104850	42.18 (U)	99.03	0.351	no	-	11	insoluble; 0.270	non-crystallizable with 0.396 confidence	Integral membrane protein
2	Chromera velia	Cvel_21181	912	99691.2	5.5	97	82	14004	145145	144270	44.87 (U)	88.06	0.066	no	-	10	soluble; 0.759	non-crystallizable with 0.495 confidence	Integral membrane protein
3	Chromera velia	Cvel_29853	770	83105.2	4.45	94	51	11715	136540	135790	41.03 (U)	100.43	0.317	no	-	12	insoluble; 0.587	crystallizable with 0.554 confidence	Integral membrane protein
4	Coprinopsis cinerea	CC1G_02731	405	43500	5.98	28	22	6107	59860	59360	34.67 (S)	98.74	0.453	no	-	12	insoluble; 0.437	non-crystallizable with 0.479 confidence	Integral membrane protein
5	Eimeria mitis Houghton	EMH_0037150	168	19410.5	9.72	17	22	2727	24980	24980	67.24 (U)	77.86	-0.336	no	-	1	insoluble; 0.329	non-crystallizable with 0.337 confidence	Secreted, cytoplasmic
6	Fusarium graminearum	FGSG_08221	541	58177.8	6.38	27	25	8195	81985	81360	29.88 (S)	100.7	0.629	no	-	12	insoluble; 0.283	non-crystallizable with 0.46 confidence	Integral membrane protein
7	Fusarium graminearum	FGSG_04240	680	75217.2	8.75	44	50	10619	148085	147710	38.25 (S)	97.16	0.38	no	-	14	insoluble; 0.413	non-crystallizable with 0.343 confidence	Integral membrane protein
8	Neospora caninum Liverpool	NCLIV_003090	1033	113756.9	8.84	69	84	15983	138670	137170	44.77 (U)	91.31	0.262	no	-	12	insoluble; 0.429	not predicted	Integral membrane protein
9	Pythium ultimum	PYU1_G009033	515	55990.6	8.6	28	34	7881	87500	86750	42.53 (U)	99.94	0.394	no	-	11	insoluble; 0.284	non-crystallizable with 0.272 confidence	Integral membrane protein
10	Saccharomyces cerevisiae	YDL210W	571	61873	6.24	41	39	8746	113635	112760	36.58 (S)	104.43	0.474	no	*	11	insoluble; 0.288	crystallizable with 0.524 confidence	Integral membrane protein
11	Vitrella brassicaformis	Vbra_175	722	76674.5	6.51	41	39	10862	167855	166730	43.47 (U)	108.16	0.608	no	-	14	insoluble; 0.262	non-crystallizable with 0.462 confidence	Integral membrane protein
12	Vitrella brassicaformis	Vbra_11331	453	48201.3	6.07	23	20	6808	110530	109780	32.18 (S)	110.11	0.744	no	SS	8	insoluble; 0.211	crystallizable with 0.543 confidence	Integral membrane protein
13	S. mansoni	353229012	494	55387.1	9.03	24	32	7873	126195	125820	27.05 (S)	109.27	0.591	no	SS	12	insoluble; 0.398	non-crystallizable with 0.371 confidence	Integral membrane protein
14	S. haematobium	MS3_01189|S	646	72717.9	8.12	44	48	10237	144950	143700	31.74 (S)	99.04	0.385	no	-	12	insoluble; 0.344	non-crystallizable with 0.41 confidence	Integral membrane protein
15	S. haematobium	MS3_06580	547	61638.4	7.81	28	30	8707	125220	124220	29.37 (S)	107.99	0.576	no	SS	11	insoluble; 0.271	non-crystallizable with 0.443 confidence	Integral membrane protein
16	S. haematobium	MS3_07417	504	56588.5	7.02	33	33	8034	107800	106800	36.32 (S)	112.78	0.561	no	-	9	insoluble; 0.232	non-crystallizable with 0.468 confidence	Integral membrane protein
17	H. sapiens	188528618	599	67073.6	8.39	36	41	9459	155575	154700	31.16 (S)	98.95	0.464	no	-	12	insoluble; 0.292	non-crystallizable with 0.433 confidence	Integral membrane protein
18	H. sapiens	21361581	602	68008.8	7.36	42	43	9571	159055	157680	38.88 (S)	98.94	0.427	no	-	12	insoluble; 0.379	non-crystallizable with 0.419 confidence	Integral membrane protein
19	H. sapiens	7657587	632	70605.8	6.52	48	46	9919	153805	152180	34.23 (S)	97.83	0.41	no	-	10	insoluble; 0.466	crystallizable with 0.501 confidence	Integral membrane protein
20	Arabidopsis thaliana	75245603	452	49856.1	8.98	25	33	7110	60320	59820	37.9 (S)	109.38	0.637	no	-	10	insoluble; 0.369	non-crystallizable with 0.388 confidence	Integral membrane protein
21	Brassica napus	923920388	450	49410.4	8.96	24	32	7066	62840	62340	26.34 (S)	112.27	0.681	no	-	10	insoluble; 0.392	non-crystallizable with 0.492 confidence	Integral membrane protein
22	Bathymodiolus septemdierum	565412058	611	68968.6	6.33	41	39	9704	150660	149660	44.04 (U)	99.87	0.406	no	-	13	insoluble; 0.302	non-crystallizable with 0.493 confidence	Integral membrane protein
23	Crassostrea gigas	405972334	673	75904.1	8.49	48	55	10709	135315	134190	32.27 (S)	98.23	0.374	no	-	14	insoluble; 0.243	non-crystallizable with 0.392 confidence	Integral membrane protein
24	Bombyx mori	953948735	580	65983.9	8.17	37	41	9303	181835	180710	37.11 (S)	100.98	0.562	no	-	13	insoluble; 0.336	non-crystallizable with 0.433 confidence	Integral membrane protein
25	Trichinella spiralis	A0A0V1B3X6	510	57783.1	6.51	41	39	8134	114540	113790	32.9 (S)	99.57	0.313	no	-	11	insoluble; 0.325	non-crystallizable with 0.427 confidence	Integral membrane protein
26	Trichinella spiralis	E5SVJ6	486	55026.7	6.51	40	38	7738	104445	103820	33.23 (S)	97.67	0.248	no	-	11	insoluble; 0.326	non-crystallizable with 0.424 confidence	Integral membrane protein

**Table 2:** Estimates of Pairwise Evolutionary Distance between Sequences.

		1	2	3	4	5	6	7	8	9	10	11	12	13	14	15	16	17	18	19	20	21	22	23	24	25	26
1	AN3304_|_Aspergillus_nidulans_FGSC_A4_|_Putative_GABA_transporter		0.108	0.111	0.117	0.237	0.098	0.108	0.096	0.127	0.11	0.102	0.123	0.127	0.103	0.1	0.102	0.103	0.108	0.111	0.132	0.13	0.103	0.112	0.105	0.137	0.138
2	Cvel_21181_|_Chromera_velia_CCMP2878_|_Na+_Cl--dependent_GABA_transporter_2	1.869		0.03	0.102	0.142	0.101	0.071	0.084	0.1	0.115	0.062	0.07	0.095	0.059	0.068	0.065	0.06	0.06	0.061	0.107	0.112	0.059	0.065	0.065	0.109	0.11
3	Cvel_29853|Chromera_velia_CCMP2878_|_Na+_Cl-dependent_GABA_transporter_2	1.94	0.517		0.103	0.142	0.097	0.071	0.086	0.098	0.106	0.064	0.071	0.096	0.06	0.066	0.065	0.057	0.057	0.058	0.112	0.11	0.057	0.063	0.06	0.114	0.114
4	CC1G_02731|Coprinopsis_cinerea_okayama7130_|_GABA_transporter	1.786	1.604	1.623		0.178	0.123	0.104	0.117	0.117	0.113	0.104	0.127	0.126	0.102	0.109	0.117	0.098	0.106	0.11	0.13	0.144	0.104	0.114	0.102	0.121	0.121
5	EMH_0037150_|Eimeria_mitis_Houghton_|_Na+_Cl--dependent_GABA_transporter_3	2.277	1.474	1.474	1.712		0.209	0.15	0.156	0.172	0.176	0.138	0.146	0.175	0.153	0.161	0.152	0.136	0.147	0.15	0.18	0.193	0.138	0.148	0.14	0.152	0.155
6	FGSG_08221|Fusarium_graminearum_PH-1_|_related_to_GABA_transport_protein	1.647	1.786	1.715	1.855	2.073		0.098	0.103	0.129	0.1	0.105	0.117	0.123	0.094	0.107	0.112	0.103	0.113	0.109	0.12	0.113	0.097	0.104	0.098	0.12	0.121
7	FGSG_04240|Fusarium_graminearum_PH-1_|_related_to_Na+_Cl--dependent_GABA_transporter_1	1.869	1.393	1.356	1.633	1.551	1.735		0.088	0.114	0.112	0.066	0.07	0.098	0.07	0.074	0.075	0.063	0.068	0.071	0.116	0.105	0.067	0.076	0.067	0.118	0.121
8	NCLIV_003090_|_Neospora_caninum_Liverpool_|_Na+_Cl--dependent_GABA_transporter_3	1.732	1.733	1.743	1.854	1.627	1.885	1.757		0.126	0.105	0.083	0.094	0.104	0.081	0.087	0.086	0.08	0.08	0.083	0.102	0.104	0.082	0.081	0.083	0.105	0.107
9	PYU1_G009033_|_Pythium_ultimum_DAOM_BR144_|_Similar_to_unc-47:_Vesicular_GABA_transporter_(Caenorhabditis_elegans)	2.027	1.689	1.68	1.743	1.755	2.103	1.917	2.181		0.13	0.115	0.129	0.135	0.11	0.105	0.11	0.111	0.109	0.109	0.128	0.116	0.107	0.115	0.107	0.139	0.144
10	YDL210W_|_Saccharomyces_cerevisiae_S288c_|_Permease_that_serves_as_a_gamma-aminobutyrate_(GABA)_transport_protein	1.863	2.009	1.893	1.747	1.786	1.74	1.968	1.955	2.151		0.119	0.135	0.122	0.103	0.109	0.114	0.094	0.103	0.102	0.131	0.116	0.101	0.099	0.1	0.133	0.136
11	Vbra_175_|_Vitrella_brassicaformis_CCMP3155_|_Na+_Cl--dependent_GABA_transporter_2	1.79	1.184	1.233	1.646	1.423	1.853	1.287	1.682	1.942	2.079		0.011	0.089	0.065	0.072	0.071	0.061	0.058	0.06	0.107	0.112	0.06	0.07	0.062	0.111	0.112
12	Vbra_11331_|_Vitrella_brassicaformis|_Na+_Cl--dependent_GABA_transporter_2	1.877	1.091	1.133	1.731	1.453	1.837	1.101	1.598	1.976	2.074	0.052		0.104	0.07	0.078	0.079	0.069	0.064	0.064	0.124	0.132	0.062	0.077	0.063	0.128	0.128
13	gi|353229012|S._mansoniputative_sodium-dependent_neurotransmitter_transporter	2.03	1.634	1.659	1.862	1.735	1.953	1.687	1.807	2.069	1.963	1.532	1.57		0.093	0.095	0.097	0.084	0.096	0.09	0.134	0.134	0.084	0.094	0.092	0.123	0.126
14	MS3_01189|S._haematobium|Na+_Cl--dependent_GABA_transporter_2	1.821	1.131	1.143	1.622	1.592	1.687	1.354	1.654	1.894	1.867	1.268	1.132	1.607		0.033	0.034	0.046	0.045	0.044	0.121	0.115	0.045	0.045	0.049	0.112	0.115
15	MS3_06580|S._haematobium|Na+_Cl--dependent_GABA_transporter_2	1.694	1.202	1.156	1.658	1.629	1.817	1.317	1.632	1.732	1.866	1.312	1.242	1.591	0.469		0.017	0.052	0.049	0.049	0.116	0.122	0.049	0.048	0.054	0.125	0.127
16	MS3_07417|S._haematobium|Na+_Cl--dependent_GABA_transporter_2	1.655	1.079	1.084	1.696	1.523	1.816	1.285	1.551	1.764	1.885	1.221	1.202	1.516	0.456	0.132		0.054	0.051	0.051	0.123	0.116	0.051	0.049	0.054	0.124	0.126
17	gi|188528618|H._sapiens|Na+_Cl--dependent_GABA_transporter_1	1.798	1.111	1.056	1.549	1.404	1.831	1.154	1.57	1.91	1.69	1.13	1.101	1.423	0.807	0.866	0.854		0.04	0.039	0.118	0.106	0.035	0.049	0.034	0.112	0.113
18	gi|21361581|H._sapiens|Na+_Cl--dependent_GABA_transporter_2	1.873	1.102	1.04	1.677	1.528	1.973	1.262	1.57	1.862	1.847	1.067	1.01	1.625	0.786	0.812	0.8	0.65		0.026	0.118	0.122	0.038	0.05	0.038	0.114	0.116
19	gi|7657587|H._sapiens|Na+_Cl--dependent_GABA_transporter_3	1.936	1.152	1.078	1.741	1.551	1.924	1.34	1.635	1.873	1.83	1.131	1.012	1.541	0.767	0.818	0.805	0.646	0.339		0.127	0.115	0.037	0.047	0.037	0.114	0.115
20	gi|75245603|Arabidopsis_thaliana|Probable_GABA_transporter_2	2.044	1.749	1.826	1.896	1.823	1.899	1.888	1.731	1.988	2.094	1.764	1.801	2.02	1.975	1.824	1.894	1.936	1.934	2.061		0.048	0.13	0.112	0.12	0.087	0.088
21	gi|923920388|Brassica_napus|GABA_transporter_1-like	2.021	1.814	1.792	2.07	1.94	1.797	1.722	1.752	1.836	1.875	1.828	1.895	2.012	1.891	1.905	1.792	1.741	1.985	1.891	0.702		0.109	0.104	0.102	0.087	0.089
22	gi|565412058|Bathymodiolus_septemdierum|GABA_transporter1	1.798	1.077	1.043	1.644	1.429	1.733	1.253	1.614	1.849	1.806	1.111	0.979	1.432	0.79	0.82	0.805	0.529	0.597	0.588	2.096	1.797		0.05	0.032	0.114	0.114
23	gi|405972334|Crassostrea_gigas|_Na+_Cl--dependent_GABA_transporter_2	1.937	1.218	1.165	1.787	1.534	1.822	1.422	1.621	1.933	1.762	1.335	1.234	1.612	0.769	0.787	0.769	0.832	0.86	0.798	1.833	1.705	0.882		0.051	0.111	0.112
24	gi|953948735|Bombyx_mori|_GABA_transporter	1.798	1.161	1.077	1.583	1.454	1.737	1.211	1.601	1.847	1.769	1.121	0.999	1.527	0.841	0.898	0.851	0.491	0.587	0.582	1.941	1.662	0.459	0.862		0.113	0.115
25	tr|A0A0V1B3X6|Trichinella_spiralis|Vesicular_GABA_transporter	2.139	1.831	1.915	1.762	1.549	1.946	1.978	1.863	2.192	2.17	1.877	1.935	1.861	1.905	1.989	1.961	1.903	1.926	1.935	1.43	1.43	1.926	1.859	1.902		0.002
26	tr|E5SVJ6|Trichinella_spiralis_|_Vesicular_GABA_transporter	2.109	1.806	1.878	1.724	1.572	1.931	1.975	1.853	2.22	2.167	1.859	1.929	1.862	1.916	1.97	1.941	1.883	1.928	1.916	1.424	1.435	1.892	1.84	1.898	0.002
